# Apparent Deviations of the Wassermann Reaction

**Published:** 1919

**Authors:** 


***Apparent Deviations of the Wassermann Reaction.*** By Dr. Georges Thibierge. *Presse Médicale*, Nov. 28, 1918.

In this article Dr. Thibierge points out that one should not expect from the Wassermann reaction more than it can yield. The biologists lead one to expect too much, hence most of the errors of interpretation.

The Wassermann reaction is not a chemical reaction; it is the research of the deviation of the complement — the research of properties of substances of unknown composition existing in unknown doses, but certainly variable, by means of other substances of equally unknown nature existing in the reagent in unknown doses, mixed in this reagent to a quantity of no less unknown substances in proportions independent from those intervening in the reaction.

Besides the antigen contained in the extract of heredo-syphilitic liver, other substances of compound or simple composition give the same reaction or emphasize it; hence have been brought about numerous modifications of the Wassermann reaction.

Errors of interpretation are incredibly frequent, partly owing to the inexperience of some chance operators who are not competent biologists.

Contrary to what some of Ehrlich's assistants thought, one cannot always make injections of salvarsan when the reaction is positive, or refrain when it is negative.

It is an absolute fact, fully recognized, that there are periods of syphilis when the reaction is negative, when, on the contrary, paludism gives a positive test.

When chancre is ignored because the reaction is not positive, the practitioner is often confronted with a fully developed roseola soon afterward. But the greatest mischief is that after such wrong prognosis the syphilitic patient is left free to disseminate contagion around him.

In the tertiary period a negative reaction may again be misleading by not revealing 10 o/o of syphilitics, carriers of manifestations in full activity.

On the other hand, a positive Wassermann will be obtained in tuberculosis, for instance, or lupus or psoriasis.

When the disease is confirmed it would be equally foolish to stop treatment because the reaction becomes negative. This would be especially harmful in the delayed affections of the nervous system.

Because of errors created by false interpretations must the practitioner be deprived of means of investigation on which to base his diagnosis?

No, but the Wassermann reaction must not be taken as error-proof, especially in the first three weeks of manifestation.

As to the marriage of syphilitics, most authorities consider there must be a period of at least four years, possibly six, after appearance of chancre, and eighteen months after last accident.

